# Clinical Assessment of Scapula Motion: Scapula Upward Rotation and Relationship with Injury in Swimmers

**DOI:** 10.3390/sports4010008

**Published:** 2016-01-28

**Authors:** Jo Brown, Rebecca Mellifont, Brendan Burkett

**Affiliations:** School of Health and Sport Sciences, University of the Sunshine Coast, Queensland 4552, Australia; jo@physiosync.com.au (J.B.); rebecca.mellifont@usc.edu.au (R.M.)

**Keywords:** dominant limb, non-dominant limb, asymmetry of motion, scapula, scapula upward rotation, injury, Paralympic swimmers

## Abstract

Abnormal scapulothoracic mechanics and scapulohumeral rhythm are implicated in shoulder pathologies, including glenohumeral impingement and rotator cuff tears. Upward scapula rotation, specifically asymmetry of scapula motion and associations of patterns through range with injury, was investigated in dominant and non-dominant limbs of nationally ranked junior and Paralympic swimmers during competition season. The static and throughout phases measures of upward scapula rotation were: Phase I (start position, 45°), Phase II (45° to 90°), Phase III (90° to 135°) and Phase IV (135° to max). Injury was assessed with a validated questionnaire. Differences between side (dominant and non-dominant), group (junior and Paralympic), and phase were examined. Significant differences (*P* < 0.05) between groups were identified for dominant side at rest, 45° and 135°, and in phases II and IV (including range). Scapulohumeral rhythm was higher in the non-dominant limb of Paralympic swimmers but in the dominant limb of junior swimmers. Greatest differences in upward rotation between injured and non-injured swimmers were found in Phase 1: 43.6% (3.3°) Paralympic; 73.1% (8°) junior. Results suggest asymmetry of movement in both limbs, through all phases, and at single points in range, should be investigated for assessing injury and developing preventive strategies and rehabilitation protocols.

## 1. Introduction

The scapula is vital in shoulder function and abnormal scapulothoracic mechanics and scapula–humeral rhythm have been implicated in shoulder pathologies, including glenohumeral impingement and rotator cuff tears. Knowledge of scapulothoracic movements is regarded as crucial in the development of preventative strategies and treatment programs in athletes with shoulder pathology [1]. In swimming, an athlete’s scapular musculature plays a pivotal role in stabilizing and preventing impingement because its continuous activation is required throughout the swim stroke [2]. Shoulder injuries have been reported in up to 90% of swimmers [3]. This high prevalence of injury in swimmers has been associated with the inability to maintain a fine balance between strength and instability, in combination with poor endurance of shoulder musculature [4]. These issues are further compounded when the asymmetry between the dominant and non-dominant arms of swimmers are compared [5,6,7]. Paralympic swimmers have reported the similar shoulder pain/injury issues as able-bodied swimmers [8], with a higher prevalence of upper limb injuries in wheelchair athletes [9]. The challenge for therapists is to accurately identify dysfunctional and/or pathological movement patterns [10], so that appropriate management strategies can be developed [11,12].

Scapula upward rotation is the dominant scapula action throughout shoulder elevation and has been previously implicated in the development of impingement [13,14]. However, inconsistencies exist in reporting the association between upward rotation and injury, particularly as recent literature supports impingement occurring below 90° abduction [11,15]. For example, some studies have found an increase upward rotation in symptomatic subjects [16], other research a decrease [17], or no difference [18,19]. This variation may be accounted for by the measurement protocols adopted, as well as the population size and type, for example, athlete *versus* non-athlete. To better understand this scapulothoracic relationship, the aim of this study was to investigate upward scapula rotation in two distinct swimming populations, with particular interest in asymmetry of scapula motion and associations of patterns through range with injury.

## 2. Materials and Methods

### 2.1. Participant Demographics

Human ethics approval was obtained prior to recruitment of participants. Forty-four athletes (22 nationally ranked junior athletes and 22 Paralympic athletes) were tested. Both groups consisted of 11 males and 11 females, and both limbs were tested in each athlete (grouped by arm dominance). The average number of years of athletes’ involvement in competitive swimming was 6 years (SD 2.6) in the junior group and 8 years (SD 3.2) in the Paralympic group. For the Paralympic group the IPC swimming class (and impairment) demographics were: S6 (short stature [3]); S7 (cerebral palsy [2], leg amputee [1]); S8 (cerebral palsy [3], leg amputee [2]); S9 (leg amputee [3], arm amputee [3]); S10 (cerebral palsy [4], arm amputee [1]). All participants were tested during the competition phase of the season. Participants were excluded if they had congenital deformity of the scapula or thorax, or had a history of trauma, fracture or surgery of the shoulder girdle.

### 2.2. Measures of the Shoulder

Two inclinometers (Plurimeter-V gravity referenced inclinometers, Fabrication, White Plains, New York, NY, USA) were used to measure upward scapula rotation, as described previously [20]. A standardized, reliable protocol was followed [21]. Reliability tests were conducted one hour apart and during three consecutive days, producing an Intraclass Correlation Coefficient of 0.95, which was similar to previous studies [22]. One examiner experienced with the use of inclinometers completed all measurements.

The inclinometers measured total shoulder abduction, which involved both glenohumeral and scapula motion within the coronal plane. Measurement required full elbow extension and neural wrist flexion and extension with the thumb leading to ensure consistent vertical alignment. The humeral inclinometer was fixed to the shaft of the humerus with Velcro straps and measured the position of humeral elevation. The scapula inclinometer was aligned along the spine of the scapula and measured upward scapula rotation. Three trials were conducted for each measure and the average score calculated.

The humeral inclinometer was fitted and checked, centered to zero and aligned so the face of the Plurimeter was vertical. The base of the scapula inclinometer was aligned along the spine of the scapula, as per previous protocols [20]. The actual resting position of each participant’s humerus relative to the vertical was recorded. Each participant performed a dynamic warm-up with arm-circles for 1 to 2 min. The participant was then asked to externally rotate the arm so that both resting humeral and scapula angles could be measured. Next, the participant was asked to move the arm through the arc of abduction, stopping at 45°, 90°, 135° and maximum shoulder abduction. The point at which motion paused for measurement was determined by the examiner with the humeral inclinometer, and resulted in five positions of abduction. At each position of abduction, the static angle of scapula upward rotation was recorded with the scapular inclinometer. The side measured was randomized and the examiner did not communicate the test results to the participant. Upward rotation was divided into four phases: Phase I (start position of 45°), Phase II (45° to 90°), Phase III (90° to 135°) and Phase IV (135° to max). By dividing the arc of motion into the four phases, the linearity of movement could be determined.

The scapulohumeral rhythm is calculated as a ratio, essentially dividing the glenohumeral elevation or abduction angle by the scapulothoracic angle (amount of upward scapula rotation). This ratio provides information about the scapula rotation, and may indicate muscle imbalance and be related to injury [17].

Shoulder pain (or injury) was defined as an incident (or occurrence) that required altered training, or rest from training, for a week or more. The swimmers who reported a change in their training of a week or more, due to pain or injury, were placed in the injury group as shown in Table 1.

### 2.3. Statistical Analysis

Means, standard deviations and 95% Confidence Intervals were calculated for all descriptive parameters of upward rotation. Differences between side (dominant and non-dominant), group (junior and Paralympic), and phase were examined with combinations of two-way ANOVA (*P* < 0.05). To provide a practical or clinical measure of any differences between these variables effect statistics were measured using Cohen’s d [23]. Both the raw difference (measured in degrees) and percentage difference were calculated so that the differences between injured and non-injured subgroups could be determined.

**Table 1:** Effect statistics comparing upward rotation measures throughout the phases of movement for injured and non-injured subgroups in junior and Paralympic groups.

	Junior Group				Paralympic Group			
Measure	Injured (°)	Non-injured (°)	Raw difference (°)	Percent difference (%)	Injured (°)	Non-injured (°)	Raw difference (°)	Percent difference (%)
	n = 15	n = 7			n = 16	n = 6		
**45°**	18.2	19.0	0.7	0%	27.4	28.8	2.7	7.5%
	(12.6 to 23.3)	(11.7 to 26.3)	(–6.6 to 8.1)	(–14.1 to 12.3)	(21 to 33.7)	(18.9 to 38.3)	(–0.2 to 14.5)	(–30.8 to 67.0)
**Phase I**	12.4	20.1	8.3	73.1%	13.4	12.8	3.3	43.6%
	(8.2 to 16.6)	(13.7 to 28.3)	(0.4 to 17.2)	(34.1 to 92.3)	(9.6 to 19.3)	(8.7 to 22.3)	(0.7 to 8.5)	(11.7 to 96.3)
**90°**	35.6	33.4	1.4	5.2%	40.8	41.3	0.7	2%
	(29.8 to 41.4)	(25.7 to 41.2)	(–8.2 to 5.3)	(–16.2 to 12.6)	(29.6 to 46.5)	(33.6 to 49.0)	(–10.8 to 12.2)	(–23.0 to 35.1)
**Phase II**	17.5	19.8	3.6	15.4%	13.7	12.5	1.2	0.8%
	(13.5 to 21.4)	(9.9 to 29.8)	(–7.5 to 14.7)	(–33.9 to 28.7)	(9.1 to 17.6)	(9.4 to 16.3)	(–5.3 to 3.0)	(–33.9 to 48.9)
**135°**	45.8	43.7	3.8	5.8%	56.9	53.0	0.7	1.2%
	(39.6 to 51.2)	(23.5 to 55.3)	(–13.9 to 6.6)	(–27.2 to 22.1)	(43.6 to 63.1)	(45.4 to 60.6)	(–8.0 to 9.4)	(–14.4 to 19.6)
**Phase III**	10.2	16.6	7.7	27.4%	16.1	13.3	1.7	1%
	(7.3 to 13.1)	(7.3 to 34.9)	(–7.9 to 23.3)	(–44.8 to 35.5)	(10.6 to 19.6)	(8.9 to 18.3)	(–7.7 to 11.0)	(–30.4 to 40.7)
**End of range**	61.9	62.6	1.3	2.2%	66.2	63.7	0.2	0.3
	(56.3 to 67.4)	(55.4 to 69.7)	(–6.6 to 4.1)	(–10.0 to 6.3)	(51.4 to 72.7)	(59.7 to 67.6)	(–6.3 to 5.9)	(–9.3 to 10.8)
**Phase IV**	19.5	32.6	12.4	42%	10.1	10.7	1.2	4.3%
	(13.6 to 25.8)	(13 to 52.1)	(–2.3 to 27.2)	(–5.4 to 25.4)	(5.5 to 11.9)	(3.9 to 17.4)	(–8.1 to 5.8)	(–25.6 to 17.1)

Note: Values are expressed as mean (95% CI).

## 3. Results

The influence of dominance on upward scapula upward rotation range and phases was investigated in both groups. Significant differences between junior and Paralympic groups were identified for the dominant side in the resting position and for upward scapula rotation at 45° and 135°, as well as at phases II and IV of the range (Table 2). No significant differences were determined between groups for non-dominant side measures.

**Table 2:** The influence of dominance on changes in upward scapula upward rotation through the range and phases of motion in junior and Paralympic swimmers.

Measure of Upward Rotation	Dominant Side		Non-Dominant Side	
	Junior	Paralympic	Junior	Paralympic
	(n = 22)	(n = 22)	(n = 22)	(n = 22)
Rest	6.3 *^,a^	14.6 *^,b^	8.8 ^a^	15.9 ^b^
	(3.8 to 8.9)	(10.0 to 18.0)	(6.0 to 14.0)	(8.0 to 17.1)
45°	18.3	27.8 *	24.7	32.5
	(14.0 to 22.0)	(22.0 to 32.0)	(18.3 to 28.0)	(20.3 to 34.0)
90°	35.6	39.0	40.5	43.2
	(30.6 to 39)	(32.0 to 45.0)	(32.0 to 43.3)	(38.0 to 46.1)
135°	45.8 *	53.5 *	54.0	56.5
	(37.9 to 49.7)	(43.0 to 60.0)	(39.0 to 56.6)	(46.0 to 61.0)
End of range°	62.1	62.5	67.7	66.5
	(58.0 to 66.2)	(54.9 to 70)	(64.4 to 70.0)	(55.3 to 70.0)
0 to 45° (Phase I)	12.4	13.3	16.0	15.0
	(8.2 to 16.6)	(10.6 to 16.7)	(12. 4 to 21.7)	(10.0 to 18.0)
45 to 90° (Phase II)	17.5 *	12.9 *	16.5	12.9
	(13.6 to 21.4)	(8.6 to 15.8)	(12.9 to 20.0)	(9.0 to 14.4)
90 to 135° (Phase III)	10.2	14.6	13.2	12.8
	(7.3 to 13.1)	(11.3 to 18.5)	(7.1 to 18.0)	(12.4 to 19)
135 to max (Phase IV)	19.5*	9.7 *	14.1	10.1
	(13.3 to 25.8)	(6.5 to 11.9)	(8.9 to 22.0)	(6.3 to 13.0)
True arc	56.8	49.2	58.9	50.5
	(52.0 to 60.0)	(43.0 to 55.0)	(53.2 to 61)	(44.0 to 56.0)

Notes: values are expressed as mean° (95% CI); significant difference (*p* < 0.05) between groups indicated by paired symbols (*/a/b).

**Figure 1:**
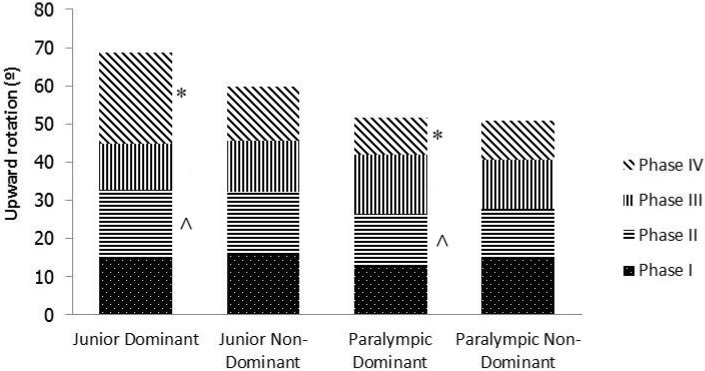
Comparison of phases of upward rotation between dominant and non-dominant limbs for junior and Paralympic swimmers. * and ^ indicate significant differences between groups with matching symbols (*P* < 0.05).

The ranges for Phase II and Phase IV differed significantly between the junior and Paralympic groups in the dominant limb only (Figure 1). In the Paralympic group, the least amount of rotation occurred in Phase IV in both limbs, whilst in the junior group the least amount of rotation occurred in Phase III in both limbs (Figure 1). This difference was reflected in a smaller rate of change of abduction and maximum upward rotation angle in the Paralympic group, and indicated a different scapulohumeral rhythm [24].

The major difference in scapulohumeral rhythm found in the Paralympic group was that the non-dominant limb had a much higher scapulohumeral rhythm than that of the dominant limb (3.5 *vs.* 2.9). However, in the junior group, this relationship was reversed, with the scapulohumeral rhythm of the non-dominant limb lower than that of the dominant limb (3.0 *vs.* 3.4). There were no significant differences between these measures of scapulohumeral rhythm.

A separate effect analysis to determine the association between injury and both phases and range of scapula upward rotation was also explored in both junior and Paralympic groups, with each group classified into injured and non-injured subgroups (Table 1). The most surprising result was that, in the junior group, a 73.1% (or 8°) difference was found between the injured and non-injured subgroups in Phase I. As there was no difference at 45°, this result most likely reflected the resting position of the scapula. Furthermore, large percentage differences were found between injured and non-injured junior swimmers in the remaining phases, resulting in 42% more range in the non-injured swimmers. For the Paralympic group, the greatest difference between injured and non-injured swimmers was 43.6% (or 3.3°) in Phase 1. For other phases, the differences between the injured and non-injured groups were probably too small for clinical relevance.

## 4. Discussion

The aim of this study was to investigate upward scapula rotation in two distinct swimming populations, with particular interest in asymmetry of scapula motion and associations of patterns through range with injury. The average total arc of scapula motion for the junior swimmers (dominant side) was 56.8°, and for the Paralympic swimmers 49.2°. These results are consistent with increased ranges of upward scapula rotation described in swimming populations and other overhead athlete populations [25] and are comparable to those found previously in injured swimmers only [20].

In the resting position, significant differences were identified between sides in the junior and Paralympic groups. The mean resting position (dominant side) found in the junior group in this study (6.3°) was similar to a previous value measured in junior swimmers: a scapula resting position angle of 5.4° was found with the arm relaxed at the side [26]. The difference identified between the groups in dominant side resting position suggests that altered scapula resting position may be a compensatory mechanism in the Paralympic group, however this speculated mechanism was not measured or quantified. That is, the altered resting position may be a direct result or influence of each swimmer’s disability on scapula mechanics, in order to maximize swim performance or muscle function for activities of daily living. It is noted this small difference between sides could also be attributed to the variability of each individual participant.

A significant difference between dominant and non-dominant sides in the mean resting scapula position (2.5° in junior and 1.3° in Paralympic groups) was found within both groups. Previously, college athletes (of similar age) have presented with values similar to those of the Paralympic group (1.46° difference) [27]. Other studies investigating scapula resting position have found no difference between those with and without impingement symptoms [19,28]. However, differing methods currently limit the usefulness of the relationship between resting scapula position and impingement symptoms. Mounting evidence suggests resting scapula position in isolation is unlikely to detect the difference between symptomatic and asymptomatic athletes [27,29], probably because asymmetry is often present in healthy individuals [30]. This combination of factors highlights the need to consider measurements of dynamic scapula asymmetry through range as part of appropriate clinical reasoning.

In the relatively stable scapula position of 45° abduction of the humerus, the significant difference between groups in upward scapula rotation (approx. 10°) may indicate poor stability resulting from muscle weakness in the Paralympic group. As previously described, surrounding soft tissue and other specific impairments in motor control or strength probably affect scapula position [30]. The significant difference also found between groups at 135° (junior group showed greater upward rotation) is potentially an adaptation to improve stroke mechanics.

Significant differences in rotation were also identified between the phases of both groups. The least amount of upward rotation occurred in Phase IV on the dominant side in the Paralympic group and in Phase III on the dominant side in the junior group. Furthermore, significant differences in upward rotation between groups in both Phase II and Phase IV were identified (Table 2, Figure 1). The largest difference occurred in Phase IV (135° to max.), a range that occupies a high percentage of the swim stroke. This difference reflects increased laxity in the shoulder joint of junior swimmers. The presence of multidirectional instability [31] has been found to cause significantly decreased upward scapula rotation up to 120°, which means that a high percentage of range occurs above this point. In the junior group, the greater rotation in the dominant limbs, with the highest portion of the range and a large between-limb difference in Phase IV (135° to max.), confirms the previous finding (Figure 1). Given established links between scapula dyskinesis and injury [11], these results have clinical implications for identifying functional, relevant dyskinesis, and for developing management strategies in these populations.

In this study the greatest difference identified between injured and non-injured subgroups within the junior group was in Phase I, with the injured subgroup demonstrating an 8.3° (73.1%) smaller range than that of the non-injured subgroup. However, at the 45° position, the range was similar in both subgroups, suggesting that this difference was due to the initial difference in resting position and the influence of soft tissue structures. This finding challenges those of previous studies that have potentially underestimated the impact of resting position in this group [27,30]. The measures of scapulohumeral rhythm (up to 3.5:1 in the Paralympic group) were larger than the common ratio of 2:1, this difference was attributed to the testing of the elite athlete population within the current study and the accepted changes in scapulohumeral rhythm associated with shoulder disorders [24]. As these results have not previously been reported in Paralympic swimming populations, no direct comparisons can be made.

Greater upward scapula rotation at 135° was found in the injured subgroups than in the non-injured subgroups (percentage differences in effect statistics of 5.8% within the junior group and 1.2% within the Paralympic group) (Table 1). However, given the small raw differences, these differences at a point in range are unlikely to be relevant. This finding contrasts with those of Su *et al.* (2004), who found athletes with impingement symptoms have demonstrated less upward rotation at 45°, 90° and 135° post swimming, a finding which is consistent with an induced fatigue effect of scapula stabilizers on the amount of scapula upward rotation. However, the athletes in our study came fresh to the data collection (*i.e.*, were not tested after swimming), and so were not influenced by fatigue, which may account for the difference in the reported results. The results further suggest that the ability to produce a consistent rate of change of scapula rotation through range, even when fatigued, should be considered a goal for swimmers. The identification of asymmetry throughout the range indicates an inability of the athlete to maintain upward scapula rotation.

Moving through the phases, effect statistics consistently showed higher percentages of upward scapula rotation range in phases III and IV in the non-injured subgroup than in the injured subgroup of the junior swimmers (differences of 27% and 42%, respectively). Compared with the injured subgroup, the non-injured subgroup had a better ability to create active range. This difference may result from underlying weakness or inhibition due to pain in the injured group [32], and hence may be a compensatory strategy. These results support the concept that the phase of motion in which the greatest range of rotation occurs, rather than the total range, is of greater clinical relevance, especially given that the range of motion through each phase reflects the balance of scapula–humeral force couples [33].

In Paralympic athletes, the greatest difference between the two subgroups (injured *vs.* non-injured) was also found in the Phase I range (43.6%). Additionally, a significant difference was found at 45°, suggesting that these results truly reflect the differences in movement that are occurring. In contrast to the junior group, a greater Phase I range was found in the injured subgroup of the Paralympic athletes. This finding also contrasts with the results of previous studies that investigated these parameters in able-bodied participants [34], which further supports the need for Paralympic swimmers to be considered as a unique group. The results may indicate use of a compensatory strategy in this group of swimmers, or even that alterations in scapula upward rotation are a transient form of scapula dyskinesis. This notion is supported by studies inducing pain [32] or dysfunction through nerve blocks [35], and by the finding that dyskinesis may be present after a single training session [36]. That 100% of athletes presented with asymmetry of one or more scapula factors in this study suggests that either a certain amount of dyskinesis is transient or that dyskinesis does not depict true dysfunction.

## 5. Conclusions

To enable an improved understanding of scapula upward rotation, asymmetry of the movement should be investigated through all phases of range, rather than at a single point in range. The phase of movement and comparison with the contra side should be considered in order to determine whether the movement is actually dysfunctional and is related to injury, or is a compensatory strategy signifying weakness of the force couple through range.

The determination of what is normal on an individual athlete basis (especially for Paralympic athletes) may be required for optimum interpretation of results, and to enable the clinician to understand asymmetry and its consequences. In addition, exact movement dysfunction must be determined to develop effective rehabilitation protocols.
